# Pain and recovery after robotic vs. uniportal lobectomy for lung cancer: a comparative analysis

**DOI:** 10.1007/s00464-025-12083-8

**Published:** 2025-08-25

**Authors:** Lubomír Tulinský, Nikol Jarošová, Dávid Adamica, Petr Bujok, Marcel Mitták, Adéla Kondé, Lubomír Martínek

**Affiliations:** 1 Department of Surgery, University Hospital Ostrava, 17.Listopadu 1790, Ostrava, Czech Republic; 2 Department of Surgical Studies, Faculty of Medicine, University of Ostrava, Syllabova 19, Ostrava, Czech Republic; 3 Central Operating Theatres, University Hospital Ostrava, 17.Listopadu 1790, Ostrava, Czech Republic; 4 Department of Informatics and Computers, Faculty of Science, University of Ostrava, 30. Dubna 22, 701 03 Ostrava, Czech Republic; 5 Department of Deputy Director for Science, Research and Education, University Hospital Ostrava, 17. Listopadu 1790, 70852 Ostrava, Czech Republic

**Keywords:** Postoperative pain, Lobectomy, Robotic-assisted thoracic surgery, Uniportal VATS, Functional recovery, Quality of life

## Abstract

**Background:**

Minimally invasive thoracic surgery techniques, such as robotic-assisted thoracic surgery (RATS) and uniportal video-assisted thoracoscopic surgery (UVATS), have revolutionized lung cancer treatment. However, comparative data on postoperative pain and functional recovery remain limited. This cohort study evaluates differences in pain intensity and pain-related activity limitations following radical lobectomy for lung cancer.

**Methods:**

A total of 140 patients undergoing lobectomy (70 RATS, 70 UVATS) were prospectively assessed. Pain intensity was measured using the Visual Analog Scale (VAS), and functional impact was evaluated with the Daily Activity Pain Interference Questionnaire (DAPIQ) on postoperative days 3 and 14. Demographic factors, including sex and body mass index (BMI), were analyzed as potential modifiers.

**Results:**

Patients in the RATS group reported significantly higher VAS scores compared to the UVATS group on postoperative day 3 (5.8 ± 2.0 vs. 3.8 ± 1.6; *p* < 0.001) and day 14 (2.7 ± 1.1 vs. 2.2 ± 1.1; *p =* 0.001). Only 17.1% of RATS patients were pain-free by day 14, compared to 34.3% in the UVATS group (*p =* 0.016). The likelihood of pain-related activity interference was 3.8 times higher in the RATS group. Female sex and lower BMI were associated with worse pain outcomes. VAS scores strongly correlated with DAPIQ results (β = 0.43).

**Conclusions:**

This study demonstrates that RATS is associated with significantly greater postoperative pain and functional limitations than UVATS following lobectomy for lung cancer. These findings underscore the importance of tailored pain management strategies in robotic thoracic surgery, particularly for higher-risk subgroups. Integration of the DAPIQ questionnaire into routine postoperative care may enhance functional recovery monitoring.

Lung cancer remains the leading cause of cancer-related mortality worldwide, with approximately 1.8 million deaths annually [1]. Surgical resection continues to be the cornerstone of curative treatment for early-stage disease, with significant evolution toward minimally invasive approaches. In contemporary thoracic surgery practice, video-assisted thoracoscopic surgery (VATS) has become standard, with uniportal VATS (UVATS) representing the least invasive approach that enables radical lobectomy with systematic lymphadenectomy through a single incision while maintaining oncological outcomes [2–4].

Concurrently, robotic-assisted thoracic surgery (RATS) has gained significant traction, offering advantages including three-dimensional visualization, tremor filtration, enhanced instrument articulation, and improved ergonomics [5]. However, the standard robotic approach using the DaVinci Xi platform necessitates four robotic ports plus an assistant port, potentially increasing chest wall trauma compared to UVATS.

A critical distinction between these approaches lies in both the number and size of incisions. The robotic technique typically requires five separate ports (most 12 mm in diameter) introduced through intercostal spaces whose anatomical width averages only 8.9 mm in the mid-axillary line at the 5th intercostal space [6]. This creates mechanical irritation and pressure on intercostal nerves, potentially increasing postoperative pain compared to the single 3–4 cm incision utilized in UVATS.

Postoperative pain significantly impacts recovery following thoracic procedures, with direct consequences for respiratory function and mobilization. Inadequately controlled pain restricts respiratory mechanics and effective coughing, increasing the risk of pulmonary complications [7]. In cancer patients specifically, prolonged recovery may delay adjuvant oncological treatment [8].

Pain interference with activities significantly impacts recovery, hindering essential components of enhanced recovery protocols like deep breathing exercises and early ambulation [9, 10]. Multiple patient factors (gender, age, BMI, psychological state) influence pain perception [11–13]. Validated tools assessing both pain intensity and functional impact are crucial for tailoring therapy and identifying patients at risk for complications [14, 15]. Despite UVATS’ theoretical advantages in reducing chest wall trauma compared to RATS, direct comparisons of these approaches regarding pain outcomes remain limited, though such data have significant implications for perioperative management.

The aim of this study is to comprehensively assess postoperative pain and its impact on recovery in patients undergoing radical lobectomy with mediastinal lymphadenectomy for lung cancer, comparing UVATS and RATS approaches. Specifically, we evaluate differences in pain intensity, character, and interference with activities during both hospitalization and home recovery, while identifying population-specific risk factors contributing to increased pain perception and functional limitations.

## Materials and methods

This study evaluated postoperative pain in patients undergoing radical pulmonary resection for lung carcinoma, comparing Robot-Assisted Thoracic Surgery (RATS) utilizing the DaVinci Xi platform with Uniportal Video-Assisted Thoracoscopic Surgery (UVATS). Secondary objectives included analyzing differences in pain intensity and character between surgical approaches relative to key demographic parameters (age > 65 years, gender, BMI > 30) and assessing pain’s functional impact on activities of daily living.

### Study population

**Fig. 1:**
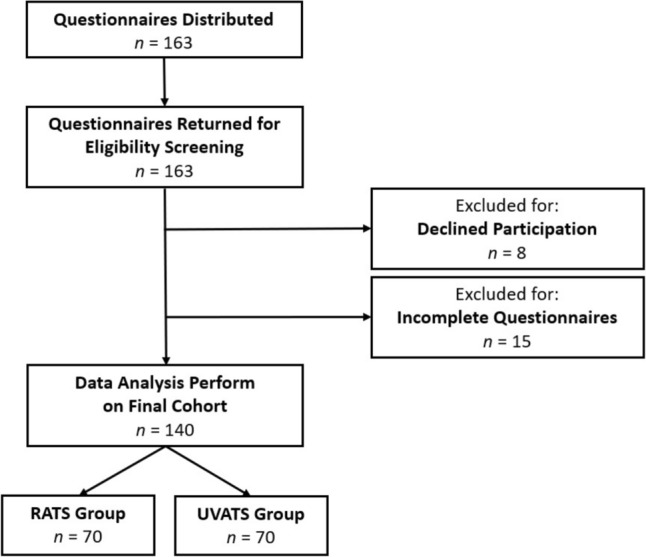
Flow diagram illustrating patient enrollment, exclusion, and group allocation based on surgical approach (UVATS vs. RATS)

The study included consecutive patients who underwent lobectomy for histologically verified lung cancer at a university hospital between October 2022 and December 2024 using either RATS or UVATS. Exclusion criteria comprised conversion to open thoracotomy, previous thoracic procedures, chronic pain requiring regular analgesics, and cognitive deficits limiting questionnaire completion. From 163 distributed questionnaires, the final cohort comprised 140 patients after excluding those who declined participation (*n =* 8) or submitted incomplete questionnaires (*n =* 15) (Fig. 1).

Patient enrollment followed a consecutive design reflecting institutional practice evolution. The UVATS cohort comprised the final 70 consecutive patients treated with uniportal thoracoscopic lobectomy through May 2023. Subsequently, our institution adopted robotic-assisted thoracic surgery as the standard approach for anatomical lung resection, and the RATS cohort included the initial 70 consecutive patients treated with this technique. All procedures were performed by the same two board-certified thoracic surgeons with equivalent experience in both techniques, employing standardized surgical approaches and instrumentation.

### Study design and data collection

Data collection employed two standardized pain assessment instruments. The Short-Form McGill Pain Questionnaire (SF-MPQ) in its validated Czech version [16, 17] allowed patients to characterize pain using descriptive adjectives with intensity ratings (0 = no pain, 1 = mild, 2 = moderate, 3 = severe), supplemented with a Visual Analog Scale (VAS, 1–10).

The Daily Activity Pain Interference Questionnaire (DAPIQ) assessed pain’s functional impact on routine activities using a six-point scale (0 = no pain, 5 = pain preventing activity). This author-modified tool was selected for its brevity, functional focus, and suitability for acute care settings compared to more extensive instruments like the Brief Pain Inventory, Pain Disability Index, PROMIS, or Multidimensional Pain Inventory (Table 1).

**Table 1:** Grading system for pain interference with daily activities according to the Daily Activity Pain Interference Questionnaire (DAPIQ)

Grade	DAPIQ Score	Clinical description
0	No Interference	Patient reports complete absence of pain
1	Minimal Interference	Pain is present but does not significantly disturb the patient; attention can be diverted from pain during activities
2	Mild Interference	Pain is present and cannot be completely ignored, but does not prevent performance of routine daily and occupational activities without errors
3	Moderate Interference	Pain cannot be ignored and interferes with routine daily activities, which are performed with difficulty and errors
4	Severe Interference	Pain is distressing to the extent that even routine daily activities can only be performed with maximum effort
5	Complete Interference	Pain is so severe that the patient is unable to perform any routine activities; forces the patient to seek relief positions or medical attention

Following ethics committee approval (No. 180/2024), patients were recruited during outpatient surgical consultations. After providing informed consent, participants completed the first questionnaire part on postoperative day 3 during hospitalization and the second part during the follow-up visit on day 14, returning completed forms in sealed envelopes for analysis. This observational cohort study was conducted and reported in accordance with the STROBE (Strengthening the Reporting of Observational Studies in Epidemiology) guidelines for cohort studies.

### Surgical technique

All procedures were performed under general anesthesia with double-lumen intubation and lateral positioning. UVATS was conducted via a single 4–5 cm incision in the fifth intercostal space. RATS involved five ports and a minithoracotomy for specimen retrieval. Standardized mediastinal lymphadenectomy was performed in all patients. Dissection and stapling devices varied according to the technique but followed institutional protocols. To reduce bias, all surgeries were performed by experienced surgeons, and no regional or neuraxial anesthesia was used perioperatively.

Postoperative pain management followed an identical standardized institutional protocol across both surgical groups. The multimodal analgesic regimen comprised intravenous paracetamol (1000 mg every 8 h) and metamizole (1000–2500 mg every 8 h) during the immediate postoperative period, with optional continuous intravenous piritramide infusion (45 mg in 45 ml normal saline at 1–3 ml/hour) titrated according to individual pain scores. As acute pain subsided, the protocol transitioned to intramuscular piritramide (15 mg as needed) combined with oral metamizole (500 mg every 8 h) and oral paracetamol (500–1000 mg every 8 h). All dosing adjustments were made based on standardized clinical evaluation and VAS scores, ensuring uniform analgesic management regardless of surgical approach.

### Statistical analysis

Statistical analyses were performed using NCSS 11 (NCSS, LLC, Kaysville, Utah, USA) and Microsoft Excel. Descriptive statistics included means with standard deviations for continuous variables and frequencies with percentages for categorical variables. Normality of continuous data was assessed using the Shapiro–Wilk test.

Between-group comparisons for continuous variables (VAS scores, age, BMI) were conducted using the Wilcoxon rank-sum test due to non-normal distribution patterns observed in pain score data. Categorical variables (DAPIQ distribution, gender, pain-free status) were compared using Pearson’s chi-square test or Fisher’s exact test when expected cell frequencies were less than five.

Linear regression analysis was employed to evaluate the relationship between pain intensity (VAS) and functional interference (DAPIQ), with standardized beta coefficients and confidence intervals reported. The probability of significant pain-related activity limitations (DAPIQ ≥ 3) was assessed using logistic regression, with odds ratios calculated for the RATS versus UVATS comparison.

Although repeated measures were collected at postoperative days 3 and 14, mixed-effects modeling was not employed due to our primary focus on discrete time-point comparisons and the study’s exploratory nature. Each time point was analyzed independently without correction for multiple comparisons. Statistical significance was set at *p* ≤ 0.05 for all analyses.

While formal multivariable regression models were not constructed for the primary outcome comparison, subgroup analyses were conducted to examine the effects of BMI and gender on pain outcomes within each surgical group. Logistic regression modeling was employed to assess the likelihood of pain-related activity limitation between surgical approaches.

## Results

A total of 140 patients were included in the study, divided into two cohorts—RATS (*n =* 70) and UVATS (*n =* 70). The mean age of all participants was 66.3 ± 8.8 years. In the RATS group, the mean age was 65.3 ± 9.8 years compared to 67.3 ± 7.6 years in the UVATS group. Regarding gender distribution, males constituted a slight majority of the total cohort (55.7%), with the RATS group having an equal gender ratio (50.0% vs. 50.0%), while males predominated in the UVATS group (61.4% vs. 38.6%). A statistically significant difference between groups was identified in BMI values (*p =* 0.011), with UVATS patients demonstrating higher mean BMI (29.3 ± 4.8 kg/m^2^) compared to RATS patients (27.4 ± 5.7 kg/m^2^). No statistically significant differences were detected between groups in other monitored demographic parameters (age, gender), as shown in Table 2.

**Table 2:** Baseline demographics of patients in the RATS and UVATS groups

	RATS, *n =* 70	UVATS, *n =* 70	Total, *n =* 140	*p* value
Age, years, mean ± SD	65.3 ± 9.8	67.3 ± 7.6	66.3 ± 8.8	0.348
Sex, n (%)				0.173
Female	35 (50.0%)	27 (38.6%)	62 (44.3%)	
Male	35 (50.0%)	43 (61.4%)	78 (55.7%)	
Body mass index (kg/m^2^), mean ± SD	27.4 ± 5.7	29.3 ± 4.8	30.6 ± 13.2	0.01

### Postoperative pain assessment by VAS on day 3 and day 14

The surgical approach was significantly associated with postoperative pain intensity. On postoperative day 3, patients undergoing robotic-assisted thoracic surgery (RATS) reported higher pain scores compared to those undergoing uniportal video-assisted thoracic surgery (UVATS) (5.8 ± 2.0 vs. 3.8 ± 1.6; *p* < 0.001). This difference remained statistically significant on postoperative day 14, with RATS patients exhibiting higher VAS scores (2.7 ± 1.1 vs. 2.2 ± 1.1; *p =* 0.001).

Age did not appear to significantly influence postoperative pain within the RATS group. The VAS scores on day 3 were 6.0 ± 2.0 for patients younger than 65 years compared to 5.7 ± 2.0 for those aged 65 years and older (*p =* 0.472). Similarly, no significant difference was observed on day 14 (2.9 ± 1.2 vs. 2.6 ± 1.1; *p =* 0.471).

Sex-related differences in postoperative pain were noted within the RATS cohort. Although no significant difference was detected on day 3 (male vs. female: 5.5 ± 2.2 vs. 6.1 ± 1.8; *p =* 0.409), a statistically significant difference was observed on day 14, with females reporting higher VAS scores than males (3.1 ± 1.2 vs. 2.5 ± 1.0; *p =* 0.019).

Body mass index (BMI) was also associated with postoperative pain outcomes. On day 3, no significant difference was found between patients with BMI < 30 and those with BMI ≥ 30 (5.7 ± 2.0 vs. 6.1 ± 2.0; *p =* 0.409). However, on day 14, patients with BMI ≥ 30 reported significantly lower pain scores (2.2 ± 0.8) compared to those with BMI < 30 (3.0 ± 1.2; *p =* 0.014). These results are summarized in Table 3.

**Table 3:** Influence of surgical technique and patient characteristics on postoperative pain at days 3 and 14

Factor	Groups	n (per group)	VAS Day 3 (mean ± SD)	*p*	VAS Day 14 (mean ± SD)	*p*
Surgical approach	RATS vs. UNI	70 vs. 70	5.8 ± 2.0 vs. 3.8 ± 1.6	< 0.001	2.7 ± 1.1 vs. 2.2 ± 1.1	0.001
Age (RATS)	< 65 years vs. ≥ 65 years	24 vs. 46	6.0 ± 2.0 vs. 5.7 ± 2.0	0.472	2.9 ± 1.2 vs. 2.6 ± 1.1	0.471
Sex (RATS)	Male vs. female	35 vs. 35	5.5 ± 2.2 vs. 6.1 ± 1.8	0.409	2.5 ± 1.0 vs. 3.1 ± 1.2	0.019
Body mass index (RATS)	< 30 vs. ≥ 30	50 vs. 20	5.7 ± 2.0 vs. 6.1 ± 2.0	0.409	3.0 ± 1.2 vs. 2.2 ± 0.8	0.014

### Postoperative pain interference by DAPIQ on day 3 and day 14

The analysis of the Daily Activity Pain Interference Questionnaire (DAPIQ) scores revealed significant differences between the RATS and UVATS groups both on postoperative day 3 (*p =* 0.013) and day 14 (*p =* 0.016).

On postoperative day 3, none of the RATS patients were pain-free (DAPIQ 0), whereas 4.3% of UVATS patients achieved this level. Minimal pain interference (DAPIQ 1) was reported in 21.4% of RATS patients compared to 32.9% in the UVATS group. Similarly, mild interference (DAPIQ 2) was noted in 25.7% of the RATS cohort and in 38.6% of UVATS patients. In contrast, higher levels of interference were more common in the RATS group: 25.7% of RATS patients were classified as DAPIQ 3 versus 10.0% in UVATS, and 21.4% of RATS patients reached DAPIQ 4 compared to 10.0% in UVATS. Complete interference (DAPIQ 5) was relatively infrequent in both groups (5.7% vs. 4.3%).

By postoperative day 14, the distribution had shifted toward lower pain interference levels in both groups. A significantly higher proportion of UVATS patients achieved a pain-free status (DAPIQ 0) compared to RATS patients (34.3% vs. 17.1%). Minimal pain (DAPIQ 1) was reported by 27.1% of RATS patients and 32.9% of UVATS patients, while mild pain interference (DAPIQ 2) remained more common among RATS patients (41.4% vs. 20.0%). Higher degrees of pain interference (DAPIQ 3 and 4) were less frequent, with 14.3% of RATS and 10.0% of UVATS patients classified as DAPIQ 3, and only two patients in the UVATS group classified as DAPIQ 4 (2.9%). No patients in either group were classified as DAPIQ 5 by day 14.

These findings suggest that patients undergoing UVATS experienced a significantly faster recovery in terms of pain interference with daily activities compared to those undergoing RATS. The detailed distribution of DAPIQ scores across both surgical approaches and assessment timepoints is presented in Table 4.

**Table 4:** Distribution of DAPIQ scores on postoperative days 3 and 14 according to surgical approach (RATS vs. UVATS)

	RATS, *n =* 70	UVATS, *n =* 70	Total, *n =* 140	p value
DAPIQ Score, Day 3, *n* (%)				0.01339
DAPIQ 0	0 (0.0%)	3 (4.3%)	3 (2.1%)	
DAPIQ 1	15 (21.4%)	23 (32.9%)	38 (27.1%)	
DAPIQ 2	18 (25.7%)	27 (38.6%)	45 (32.1%)	
DAPIQ 3	18 (25.7%)	7 (10.0%)	25 (17.9%)	
DAPIQ 4	15 (21.4%)	7 (10.0%)	22 (15.7%)	
DAPIQ 5	4 (5.7%)	3 (4.3%)	7 (5.0%)	
DAPIQ Score, Day 14, *n* (%)				0.01632
DAPIQ 0	12 (17.1%)	24 (34.3%)	36 (25.7%)	
DAPIQ 1	19 (27.1%)	23 (32.9%)	42 (30.0%)	
DAPIQ 2	29 (41.4%)	14 (20.0%)	43 (30.7%)	
DAPIQ 3	10 (14.3%)	7 (10.0%)	17 (12.1%)	
DAPIQ 4	0 (0.0%)	2 (2.9%)	2 (1.4%)	
DAPIQ 5	0 (0.0%)	0 (0.0%)	0 (0.0%)	

**Fig. 2:**
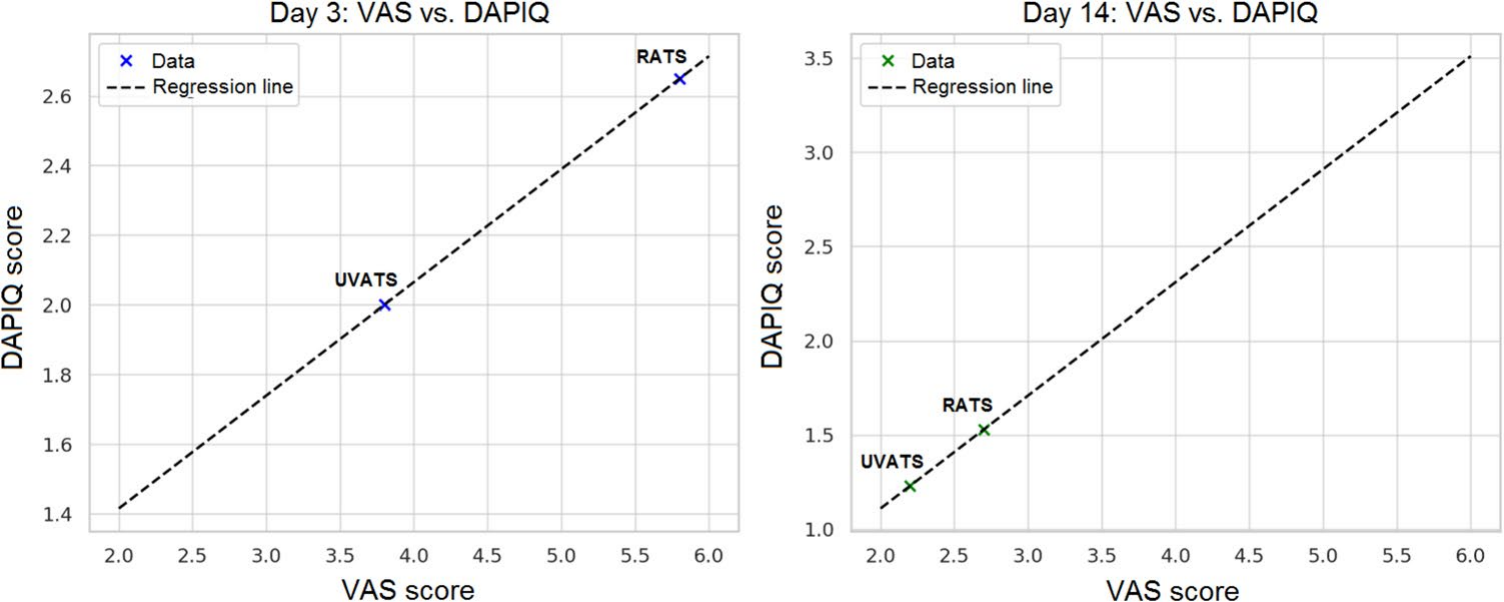
Correlation between pain intensity (VAS) and daily activity interference (DAPIQ) scores on postoperative days 3 and 14. As illustrated, higher VAS scores were associated with increased DAPIQ scores, indicating greater interference of pain with daily activities, particularly in patients who underwent RATS. This relationship was evident both on postoperative day 3 and day 14.

Based on the collected data and regression analysis performed, two visualizations were created illustrating the relationship between pain intensity (VAS score) and the degree of pain interference with daily activities (DAPIQ score) on postoperative days 3 and 14. The graph for postoperative day 3 confirms that higher VAS scores (observed in RATS patients) are demonstrably associated with higher mean DAPIQ scores—indicating greater limitation of daily activities (Fig. 2). The graph for postoperative day 14 confirms that this trend persists after two weeks, albeit with a milder slope of declining correlation (Fig. 2).

This objectively demonstrates that higher pain intensity in RATS patients is associated with impairment of normal functioning, as evidenced by the statistically significant difference in DAPIQ and its positive correlation with VAS.

Clinical interpretation: Each additional point on the VAS corresponds to an increase in DAPIQ by 0.43 points. RATS patients with VAS > 5 have a 3.8 × higher probability of DAPIQ ≥ 3 compared to UVATS patients. This analysis objectively demonstrates that higher pain (VAS) in the RATS group indeed correlates with more significant limitations in daily activities (DAPIQ). These findings validate the DAPIQ as a reliable instrument for assessing the interaction between postoperative pain and functional capacity in thoracic surgery patients, demonstrating strong correlation with established pain intensity measures.

## Discussion

Our study was designed to provide an objective comparison between robotic-assisted thoracic surgery (RATS) and uniportal video-assisted thoracoscopic surgery (UVATS) for radical lobectomy with mediastinal lymphadenectomy. By employing standardized assessment tools and excluding regional analgesic techniques, we ensured that observed differences in pain intensity and functional limitations were attributable to the surgical approach rather than variations in analgesic management.

### Assessing the functional impact of postoperative pain

The Daily Activity Pain Interference Questionnaire (DAPIQ) was selected for its concise, targeted assessment of how postoperative pain affects essential daily activities. Unlike comprehensive tools developed for chronic pain settings, DAPIQ is designed for the acute, fluctuating nature of postoperative pain, allowing efficient monitoring in patients with limited stamina during early recovery [18–20].

Contemporary guidelines emphasize assessing not only pain intensity but also its impact on functional recovery, as pain interference with activity is often more clinically significant than pain at rest [18, 20, 21]. While instruments like the Brief Pain Inventory provide comprehensive coverage, their length may be impractical for repeated use in the immediate postoperative period [18]. DAPIQ’s single-item format minimizes patient burden while directly quantifying pain’s interference with daily activities, aligning with the concept that functional recovery is a key outcome of postoperative pain management.

Our findings regarding the correlation between pain intensity and functional interference complement those of Bendixen et al. (2022), who demonstrated that patients with higher pain scores during the first postoperative week experienced significantly delayed recovery of physical function [22]. The observed difference in DAPIQ scores suggests that UVATS is associated with more favorable early recovery, with twice as many pain-free patients by day 14 compared to RATS. This may support earlier return to daily activities or initiation of adjuvant therapy when indicated.

Functional recovery represents a key component of postoperative quality of life in thoracic surgery [23]. The DAPIQ questionnaire allowed broader evaluation of pain’s functional impact than traditional pain scales, offering valuable insight into how surgical technique affects patients’ daily function during early recovery.

Although this study focused on pain intensity and its interference with functional recovery, we also monitored the incidence of postoperative pneumonia, which was 10.8% across the cohort, with no significant difference observed between the RATS and UVATS groups. However, the study was not designed or powered to evaluate the relationship between pain and pulmonary complications such as atelectasis or infection. Further prospective research is currently underway at our institution to explore this association in greater depth, including the role of pain intensity, regional analgesia, and perioperative bronchoscopy. These data will help clarify whether elevated pain levels in the early postoperative period translate into clinically relevant respiratory complications.

### Pain Intensity comparison between RATS and UVATS

Our study confirmed significantly higher postoperative pain in patients undergoing RATS compared to UVATS, with the greatest difference observed on day 3 (VAS 5.8 vs. 3.8; *p* < 0.001), persisting through day 14. Despite the technical advantages of robotic systems, these findings suggest that RATS may be associated with greater pain burden, potentially impacting early recovery.

A plausible explanation for higher pain intensity in RATS may be attributed to the number, size, and fixed positioning of ports required by the robotic system architecture. These ports may cause more extensive intercostal nerve trauma compared to the single port utilized in UVATS. Additionally, the relative rigidity of robotic instrumentation may exert greater tensile forces on the chest wall structures. This hypothesis aligns with the physiological mechanism of intercostal pain following thoracotomy described by Wildgaard et al. [8]. Our results are consistent with the findings of Novellis et al. (2021), who reported higher pain intensity in patients undergoing RATS compared with VATS at 14 weeks (*p =* 0.004) and 12 months after surgery (*p =* 0.02) [24].

Recent research further complicates this landscape by reporting comparable pain scores between RATS and multiportal VATS. Several studies, including randomized controlled trials, have documented equivalent pain scores between RATS and VATS in the initial postoperative period and at three to six months after surgery [25, 26]. This contrasts with our findings, which demonstrate significantly higher pain scores in patients undergoing RATS compared with those treated with UVATS.

Retrospective observational data have generally shown no significant differences in long-term pain between RATS and VATS cohorts, although some evidence suggests that RATS may be associated with inferior quality of life outcomes [27]. A post hoc analysis of the RVlob randomized trial [28] reported largely equivalent results between RATS and VATS across most domains, with the notable exception of elevated pain scores in the RATS group at four weeks postoperatively. In certain cohorts, the prevalence of chronic pain was marginally elevated in the RATS group; however, these disparities did not attain statistical significance. These findings underscore the complexity of postoperative pain assessment and the necessity of differentiating between acute and chronic pain when evaluating surgical techniques [29, 30].

The variability across existing studies reflects the complexity of postoperative pain assessment, influenced by surgical technique, patient factors, and evaluation methods. Our findings add to this evidence by directly comparing RATS and UVATS, revealing a potentially meaningful difference in early pain severity. Future randomized studies focusing on pain as a primary outcome and accounting for technical nuances are needed to clarify these disparities.

### Age-related differences in pain perception

Our analysis demonstrated that patient age did not significantly influence postoperative pain intensity following lobectomy. This finding contradicts the widely held belief that older patients experience lower pain intensity due to higher pain thresholds. Lautenbacher et al. (2017) in their meta-analysis [31] reported that pain threshold increases with age, consistent with findings by Šnircová et al. (2007), who observed lower pain incidence in older patients [32].

The absence of age-related differences in our study may be attributed to the implementation of a more sophisticated standardized analgesic protocol or may suggest that responses to surgical stress and postoperative pain are more complex than simply age-related changes in pain threshold. Gibson and Helme (2001) propose that age-related changes in pain perception may be offset by alterations in pharmacokinetics and pharmacodynamics of analgesics in elderly patients, resulting in comparable subjective pain experiences despite physiological differences [33]. Furthermore, Gagliese et al. (2008) observed that the correlates of postoperative pain intensity and opioid consumption differ between younger and older patients, and that the same factors may have distinct effects across age groups. Their findings suggest that age-specific profiles of pain and analgesia responses contribute to the lack of a straightforward relationship between age and clinical pain ratings [34].

### Gender-specific pain responses

Sex-related differences in postoperative pain were observed primarily within the RATS cohort. While no significant difference was found on day 3 (male vs. female: 5.5 ± 2.2 vs. 6.1 ± 1.8; *p =* 0.409), females reported significantly higher VAS scores by day 14 (3.1 ± 1.2 vs. 2.5 ± 1.0; *p =* 0.019). This aligns with previous studies showing greater postoperative pain intensity and longer duration in women [35, 36].

These differences have been attributed to hormonal influences, pain modulation pathways, and psychosocial factors [35–37]. Notably, our findings echo observations by Fillingim et al., who reported that gender differences become more apparent during subacute recovery phases rather than immediately after surgery [37]. This suggests that women may have a slower decline in pain or a higher risk of persistent postoperative pain.

Analgesic consumption patterns further supported this trend, with female patients more frequently requiring combined or prolonged oral analgesia. These results are consistent with reports by Šnircová et al. and Mills et al., who demonstrated both greater maximum pain scores and lower pain thresholds among female patients [32, 38].

Our data reinforce the need for sex-sensitive pain management protocols following lobectomy, particularly in robotic-assisted procedures, and highlight the relevance of tailored approaches to address persistent pain disparities beyond the early postoperative period.

### BMI impact on postoperative pain

The influence of BMI on postoperative pain after RATS was modest but significant in our cohort, with patients having BMI < 30 reporting higher pain values than those with higher BMI (3.0 vs. 2.2; *p =* 0.01386). This result contradicts some published studies, such as Yang et al. (2019), who identified higher BMI as a predictor of more intense postoperative pain [39]. Conversely, McElnay et al. (2014) found no significant relationship between BMI and postoperative pain following VATS [40].

This unexpected association may be due to biomechanical or physiological factors. A greater amount of adipose tissue may protect the thoracic nerves from mechanical irritation during robotic surgery, while reduced mobility in patients with a higher BMI may limit tension at the incision site. Differences in inflammatory or pain mediator responses may also play a role. However, BMI is a crude indicator that does not reflect fat distribution or tissue composition. Our findings should be interpreted with caution, and future studies using advanced body composition analysis are needed to better understand these associations.

Although subgroup analyses were conducted to examine the effects of sex and BMI on pain outcomes, we acknowledge that multivariable regression analysis adjusting for these baseline differences was not performed in our primary group comparison. However, the pain intensity differences between RATS and UVATS remained consistent across demographic subgroups, and logistic regression confirmed a significantly higher likelihood of pain-related functional interference (DAPIQ ≥ 3) in the RATS group. We recognize that future studies employing multivariable modeling or propensity score matching would provide more robust control for potential confounders and enhance the precision of surgical approach comparisons.

### Study limitations

Our study has several limitations. First, the follow-up period (14 days) captures early recovery but not long-term outcomes or chronic pain development. Second, while our sample size was adequate for primary analyses, subgroup evaluations may have been underpowered. Third, we focused on pain and functional interference without assessing other quality of life domains or objective functional measures. Fourth, we did not systematically collect quantitative data on total opioid consumption or calculate morphine equivalent doses, which would have provided additional objective measures of pain management effectiveness alongside our patient-reported outcome instruments. Fifth, our statistical approach analyzed each time point independently rather than employing mixed-effects models, which would have better handled repeated measurements and individual patient trajectories while reducing potential Type I error from multiple comparisons.

Future research should include (1) extended follow-up to 3, 6, and 12 months to assess chronic pain development, (2) randomized controlled trial design comparing UVATS and RATS with pain as the primary outcome, (3) integration of objective functional assessments (e.g., pulmonary function tests, 6-min walk test), (4) evaluation of specific technical modifications to the robotic approach that might reduce pain, and (5) investigation of tailored analgesic protocols accounting for both surgical approach and patient-specific factors identified in this study.

In conclusion, patients undergoing RATS for radical lobectomy have significantly more postoperative pain and lower functional recovery than patients treated with UVATS. RATS patients were 3.8 times more likely to report significant pain-related activity limitations. These results highlight the impact of the surgical approach on early quality of life and underscore the need for improved pain management in robotic thoracic surgery, especially in higher-risk groups such as women. The DAPIQ questionnaire proved to be a practical tool for assessing postoperative functional impact and should be integrated into routine care to support personalized recovery strategies.
